# Use of Phage Display technology in development of canine visceral leishmaniasis vaccine using synthetic peptide trapped in sphingomyelin/cholesterol liposomes

**DOI:** 10.1186/s13071-015-0747-z

**Published:** 2015-02-28

**Authors:** Christina Monerat Toledo-Machado, Lilian Lacerda Bueno, Daniel Menezes-Souza, Ricardo Andrez Machado-de-Avila, Christophe Nguyen, Claude Granier, Daniella Castanheira Bartholomeu, Carlos Chávez-Olórtegui, Ricardo Toshio Fujiwara

**Affiliations:** Departamento de Parasitologia, ICB, Universidade Federal de Minas Gerais, CP: 486 - CEP: 31.270-901, Belo Horizonte, Minas Gerais Brazil; Departamento Bioquímica e Imunologia, ICB, Universidade Federal de Minas Gerais, CP: 486 - CEP: 31.270-901, Belo Horizonte, Minas Gerais Brazil; Unidade Acadêmica de Ciências da Saúde, Universidade do Extremo Sul Catarinense - CEP: 88.806-000, Criciúma, Santa Catarina Brazil; SysDiag CNRS-BioRad UMR 3145, Cap Delta/Parc Euromédecine, 1682 rue de la Valsière, CS 61003, 34184 Montpellier Cedex 4, France

**Keywords:** Phage Display, Canine Visceral Leishmaniasis, Vaccine, Synthetic Peptides, Liposomes

## Abstract

**Background:**

*Leishmania* parasites can cause visceral or cutaneous disease and are found in subtropical and tropical regions of the Old and New World. The pathology of the infection is determined by both host immune factors and species/strain differences of the parasite. Dogs represent the major reservoir of *Leishmania infantum* (syn. *L. chagasi*) and vaccines are considered the most cost-effective control tools for canine disease.

**Methods:**

Selection of immunodominant peptides was performed by Phage Display to identify sequences recognized by *L. infantum* naturally infected animals. Sera from *Leishmania* infected animals were used in the biopanning to selection of specific peptides. Serum samples from *T. cruzi* infected and healthy animals were used as control. After selection, synthetic peptides were produced in membrane (spot-synthesis) in soluble form and blotting and ELISA were performed for validation of serum reactivity. Selected peptide was formulated with aluminum hydroxide and liposomes and immunization was performed in BALB/c mice. Protection was determined by qPCR after challenge infection with virulent *L. infantum*.

**Results:**

We reported the selection of Peptide 5 through Phage Display technique and demonstrate its ability to promote a state of immunity against *L. infantum* infection in murine model after immunization using liposomes as vaccine carrier. Our results demonstrate that immunization with Peptide 5 when formulated with aluminum hydroxide and liposomes is immunogenic and elicited significant protection associated with the induction of mixed Th1/Th2 immune response against *L. infantum* infection.

**Conclusion:**

Peptide 5 is a promising vaccine candidate and the findings obtained in the present study encourage canine trials to confirm the effectiveness of a vaccine against CVL.

## Background

Canine visceral leishmaniasis (CVL), caused by *Leishmania infantum*, is a endemic disease and affects millions of dogs all over the world such as the Mediterranean basin, China, Latin America and is an emergent in North America [1,2]. Transmission between dogs, or from dogs to human occurs by the bite of a phlebotomine sand fly. Dogs are the main reservoir and responsible for maintaining the domestic cycle of parasites while stray dogs and wild canids maintain the peridomestic cycle [3,4]. Control of CVL is proposed to be central for limiting the endemic transmission cycle of this zoonotic disease [5,6]. There are evidences that *Leishmania*-infected dogs never achieve parasitological cure, and the widespread use of the available anti-*Leishmania* drugs for human and canine treatment may contribute to parasite drug resistance [7,8].

Strategies to control CVL are still ineffective [9]. Vaccination has proven to be the most effective way to reduce mortality and morbidity caused by other infectious diseases in the history of humanity. The development of an effective vaccine in dogs would restrict the spread of the disease reducing infectivity to sand fly vectors and transmission to human beings [10,11]. Among the different approaches for vaccine development, Phage Display proved to be a successful technique due to fast identification of molecules that might be useful in the design of new immunotherapeutic agents and vaccines [12-14]. In the current work, we demonstrate that a selected peptide (Peptide 5) using Phage Display technique, when formulated with Aluminum Hydroxide and Cholesterol/Sphyngomielin liposomes, is highly immunogenic and presented a significant protective effect in murine model after challenge infection with virulent *L. infantum*. Taken together, our results show that Peptide 5 is a promising vaccine candidate and support CVL control.

## Methods

### Production of total *L. infantum* protein antigen (LiAg)

*L. infantum* (MHOM/BR/1975/BH46) were grown at 24°C in Schneider’s medium (Sigma-Aldrich) supplemented with 10% fetal bovine serum (Cultilab, Brazil), 200 U/mL penicillin and 100 μg/mL streptomycin (all from Life Technologies, USA), pH 7.2. Total crude antigen of *L. infantum* (LiAg) was prepared from stationary phase promastigotes, submitted to 7 cycles of freezing (liquid nitrogen) and thawing (42°C), followed by cell disruption by sonication (Ultrasonic processor, GEX600) with cycles of 10 sec for 2 min at 35 MHz. The extracts were then submitted to centrifugation at 8,000 *g*, 20 min at 4°C. Supernatant was collected and stored at −80°C until further use. The protein concentration was estimated by the Bradford method [15].

### Preparation of antibodies for Biopanning

Antibodies used for biopanning were purified from 38 sera from *L. infantum* naturally infected dogs. Infection was determined by a positive immunofluorescence (IFAT) titre at a 1:40 serum dilution, positive reactivity in ELISA and parasitological diagnosis of *Leishmania* in bone marrow or spleen samples. Antibodies from sera of 38 healthy dogs with negative IFAT and parasitological tests were included as negative controls. Fifteen serum samples of dogs experimentally infected with *Trypanosoma cruzi* parasite were also collected. Polyclonal IgGs from infected animals specific to LiAg were purified using ammonium sulfate precipitation and filtration through ProteinA-Sepharose-4B column [16]. Following elution and neutralization using NaOH 0.1 M, the IgG fraction was dialyzed with PBS and the protein concentration was determined by the Bradford method [15]. IgGs from negative controls and from *T. cruzi* infected dogs were also fractionated as described. IgG reactivity against LiAg was confirmed by ELISA.

### Ethics statement

All sera samples used for biopanning were obtained from the Veterinary Hospital of the Federal University of Minas Gerais and the experiments were performed in compliance with the University’s Animal Experimentation Ethic Committee (CETEA), protocol 122/2009. All sera were stored at −20°C until use. Procedures related to immunization were also approved by CETEA (protocol 44/2012).

### Phage display experiments

#### Biopanning

M13 phage libraries expressing 15-mer (*X*_15_) and 12-mer peptides (*X*C*X*_8_C*X*) were described by Bonnycastle et al. (1996) [17] and obtained from Dr. John Scott (British Columbia, Canada). Three cycles of biopanning were performed as described previously [18]. After three rounds of enrichment, individual phage clones were isolated and further analyzed.

#### Screening

ELISA plates (Becton Dickinson) were coated with 1 μg/well of anti-LiAg IgGs or anti-*T. cruzi* IgGs in 100 mM NaHCO_3_, pH 8.6 and overnight at 4°C. Plates were washed with PBS, 0.1% Tween 20 and then blocked with PBS, 0.1% Tween 20 2% non-fat dried milk for 1 h at 37°C. 10^10^ TU of individual phages isolated after third enrichment step and 50 μL of blocking buffer was then added to each well. Phage particles were incubated for 2 h at 37°C. Binding was detected using a peroxidase conjugated anti-M13 antibody (Roche) diluted 1:3000 in blocking buffer. After 1 h at 37°C and washing, the peroxidase substrate was added. Resulting color was measured at 492 nm with an automated microtiter plate reader (Bio-Rad, USA). Afterwards, twelve clones were selected and checked by ELISA for their ability to bind to anti *T. cruzi* dog IgG.

#### Phage binding analysis by ELISA

The clone selected with anti-LiAg antibodies was used in a new format-ELISA to verify their reactivity with sera samples of dogs with VL. Plate was sensitized with 10^10^ phages/mL of individual clones or with LiAg (0.5 μg/well) in coating buffer. The plate was washed and blocked as previously described and incubated with 100 μL of sera samples of infected and healthy dogs, diluted 1:100, in PBS-Tween 20 (0.05%) for 2 h at 37°C. Binding was detected using a peroxidase conjugated anti-dog IgG antibody (Sigma-Aldrich) diluted 1:5,000 in blocking buffer. After 1 h at 37°C and washing, the peroxidase substrate was added and reaction was measured as previously described.

### DNA sequencing, synthesis, chromatography and mass spectrometry of soluble peptides

Approximately 9 μg of single-stranded DNA was purified using the QIA prep Spin M13 protocol (Qiagen). Sequencing reactions were carried out according to the dideoxy chain termination method [19], using the ABI Prism Kit (Applied Biosystems) for the automatic method with ABI PRISM 377 (PerkinElmer). The primer reverse 5’-TCGGCAAGCTCTTTTAGG-3’ was used for sequencing. The sequence obtained was translated.

### Peptides synthesis on cellulose membrane

Peptide selected for immunization and a non-related peptide were synthesized on a cellulose membrane, as previously described by Laune et al. (2002) [20]. Membranes were obtained from INTAVIS (Koln). An ASP222 robot (Intavis) was used for the coupling steps. Peptide was acetylated at the N-terminus. After the peptides sequences had been assembled, the side-chain protecting groups were removed by trifluoroacetic acid treatment [21]. After peptide synthesis on cellulose membrane, ELISA-Spot was performed. Membranes were washed 3 times with TBS (Saline, KCl 0.002 M, Tris 0.05 M) pH 7.4 and blocking solution was added (casein 0.5% and T-TBS 0.05%). After sixty minutes, membranes were incubated with positive and negative canine serum pool 1:100. After 1 h at 37°C and washing, the anti-dog peroxidase conjugate was added. After incubation for 30 min with substrate (MTT-BCIP), membranes were analyzed according to the resulting color.

### Synthesis of soluble peptide

Soluble peptides were synthesized in ResPep SL Synthesizer by Fmoc chemistry [22]. After synthesis the peptides were desprotected and released from the resin by trifluoroacetic acid treatment in the presence of the appropriate scavengers. The peptide was lyophilized and their purity assessed by HPLC and their mass confirmed by mass spectrometry according to Machado-de-Avila et al. (2011) [23].

### Preparation of liposomes containing peptide

Immunogens (selected peptide – Peptide 5 or LiAg) and PBS (as control) were encapsulated in liposomes, which were used as vaccine carrier. Sphingomyelin/Cholesterol multilamellar liposomes (ratio of 2:1) were prepared by dissolving 25 mg of sphingomyelin (Sigma-Aldrich) and 6.5 mg cholesterol (Sigma-Aldrich) in 20 ml chloroform together with traces of methanol. The solution was kept in a 1,000-ml round-bottom flask and the solvent was removed by flash evaporation on a rotary evaporator at 37°C. After drying under reduced pressure for 80 min, the aqueous phase containing 2.1 mg of immunogens in PBS, pH 7.4 was added to the flask. The lipid film was dislodged from the glass by the use of a vortex mixer. The liposomes were retrieved using a Pasteur pipette and then treated with ultrasonic vibration three times during 20s each. The liposome suspension was centrifuged at 8,000 *g* for 10 min at 4°C to remove non-encapsulated immunogens and the supernatant protein concentration was estimated by spectrophotometry. The encapsulation efficiency for the formulations was 85%. The pelleted liposomes were resuspended and washed three more times with PBS by centrifugation and 0.5 mg Aluminum Hydroxide was added (200 μg/animal). The compound was stored in PBS at 4°C.

### Immunization of Balb/c with Peptide 5 formulated into liposomes

Mice were divided into 3 groups (18 mice/group). Each group was intraperitoneally immunized (Day 0) as follows: group I, liposomes that entrap the selected peptide (Peptide 5) (50 μg/100 μL, intraperitoneal); group II, liposomes that entrap LiAg (50 μg protein/100 μL intraperitoneal); group III, liposomes that entrap PBS (100 μL, intraperitoneal). On Day 15, mice were boosted with the same immunogen at an equivalent dose. Mice were again immunized on days 22, 29, 36 and 43. Two weeks after last immunization (dasy 58), nine mice of each group were euthanized and spleens were harvested for cytokine measurements. Challenge infection on remaining animals (9 per group) was performed seven days after last immunization (day 65). Mice were inoculated intraperitoneally with 1 × 10^6^ *L. infantum* stationary promastigotes and after 4 weeks the parasite burden was determined by qPCR. Mouse β-actin gene (F: CAGAGCAAGAGAGGTATCC; R: TCATTGTAGAAGGTGTGGTGC) was used as internal control in order to confirm DNA integrity and to verify qPCR inhibitors (data not showed). In this step, β-actin qPCR was performed in parallel for each sample. As additional controls, five non-immune animals were also infected.

### Evaluation of immunogenicity induced by vaccination

Antibody production was determined by indirect ELISA. Briefly, ELISA plates (Becton Dickinson) were coated with 2 μg/well of Peptide 5 or LiAg (0.5 μg/well) in PBS and further blocked with PBS with 0.1% Tween 20 and 5% BSA at 37°C. After 1 h *pool* of serum were serially diluted up to 10^7^. Binding was detected using a peroxidase conjugated anti-mouse antibody (Roche) diluted 1:5,000 in incubation buffer. After 1 h at 37°C and washing, the peroxidase substrate was added. The resulting color was measured at 492 nm with an automated microtiter plate reader (Bio-Rad). Endpoint titers were assigned by taking the lowest dilution with an OD that was higher than the negative control plus 0.100 [24].

In order to evaluate cellular response, spleen samples were obtained from immunized animals. Splenocytes were resuspended in RPMI 1640 containing 10% FBS (Sigma-Aldrich) and seeded at 1×10^6^/mL in 48-well flat-bottom plates (Nunc). The spleen cells were stimulated *in vitro* with either Pep 5 (l0 μg/well) or LiAg (1 μg/well) or PHA (25 μg/mL) as a positive control for viability and cytokine production, and incubated at 37°C in 5% CO_2_ for 48 h and then the supernatants were collected. The level of IL-10, IFN-γ, TNF-α and IL-4 were determined by sandwich ELISA kit according to the manufacturer’s instructions (R&D systems). Cytokine concentrations were calculated from the standard curve using 5-parameter curve fitting software (SOFTmax®Pro 5.3, Molecular Devices).

### Extraction of DNA and evaluation of spleen parasite load by qPCR

DNA was extracted from the spleen samples using NucleoSpin®Tissue (Macherey-Nagel) according to the manufacturer’s instructions. The parasite load was calculated by qPCR according to a method described elsewhere [25,26] with minor modifications. The parasite burdens were estimated using the following primers: Forward, 5’-TGTCGCTTGCAGACCAGATG-3’ and Reverse, 5’-GCATCGCAGGTGTGAGCAC-3’. These primers amplified a 90 bp fragment of a single-copy-number *L. infantum* DNA polymerase gene (GenBank: AF009147). PCR was carried out in a final volume of 10 μL containing 2 pmol of each DNA polymerase primers, SYBR®Green (Applied Biosystems), 4 μL of DNA with a concentration of 5 ng/μL and enough volume of ultrapure water. Reactions were processed and analyzed in an ABI Prism 7500 Sequence Detection System (Applied Biosystems). The following steps were programmed: 95°C for 10 min followed by 40 cycles at 95°C for 15 s and 60°C for 1 min. Parasite quantification for each spleen sample was calculated by interpolation from the standard curve included in the same run, performed in duplicate, and expressed as the number of parasites per 20 ng total DNA.

### Statistical analysis

GraphPad Prism 5 (GraphPad Inc., USA) was used for statistical analysis. The Kolmogorov-Smirnov test was used to verify data distribution and Grubb’s test was used to detect the outliers in the samples. Mean values of normally distributed data were compared using one-way analysis of variance (ANOVA) and P-values were assessed using Tukey’s post-hoc analysis. Differences of P < 0.05 were considered significant.

## Results

### Phage reactivity by ELISA and mimotope peptide synthesis

**Figure 1:**
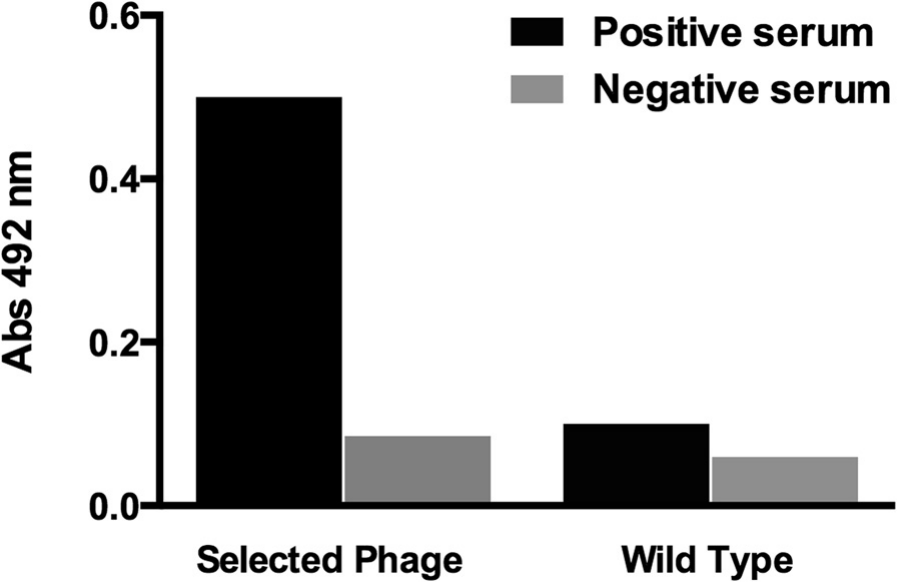
**Reactivity of mice serum against phage display selected clones.** Plate was sensitized with 10^10^ phages/mL of individual clone and *Wild type* phage (negative control). Positive and negative dog serum were employed (black and gray bars, respectively). Binding was detected using a peroxidase conjugated anti-dog IgG antibody. Absorbance values at 492 nm were means of duplicates. The results are expressed as mean of the group (bars).

In order to identify peptides that bind to anti-LiAg antibodies, four different phage libraries were screened. A significant enrichment of phage binding to the target antibodies was obtained after three rounds of panning. One hundred and ninety-eight phage clones were randomly picked from the third round of selection, and twelve clones were selected based on their reactivity (absorbance at 492 nm ≥ 1.0) against IgGs from *L. infantum* infected dogs. Afterwards, twelve clones were checked by ELISA for their ability to bind to anti-*T. cruzi* dog IgGs. Clone 5 (originating the Peptide 5) was further selected due to the highest reactivity against *Leishmania-*specific IgGs and any cross-reactivity with IgGs from dogs with Chagas disease (data not shown). Figure 1 shows the reactivity of sera samples of dogs with VL when the individual clone (Peptide 5) was used to sensitize the ELISA plates.

The DNA sequence of clone 5 was translated and the amino acid sequence of the peptide was deduced (ICARQDPAGNCS). The corresponding synthetic peptide was chemically synthesized, purified by reverse phase chromatography and its correct molecular weights were confirmed by mass spectrometry.

### ELISA-Spot assay

**Figure 2:**
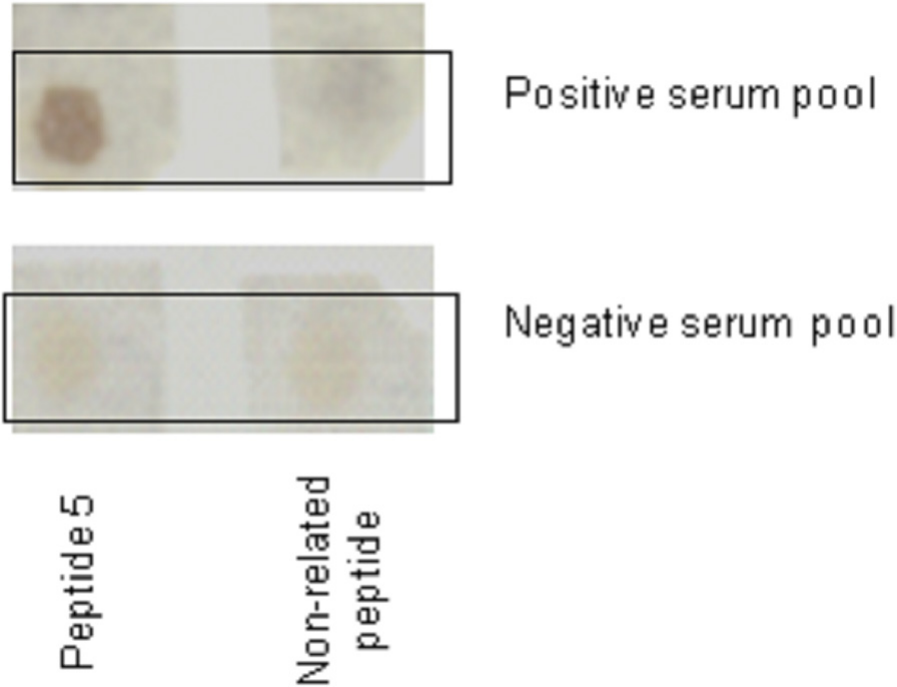
**Reactivity of peptide selected by phage display on a SPOT membrane.** Membrane containing Peptide 5 sequence was incubated with positive and negative canine serum pool at a dilution 1:100. Assay employing non-related peptide was used as reaction control. Reaction was detected using peroxidase conjugated anti-dog antibody (1:5000).

Peptide 5 (corresponding to the sequence ICARQDPAGNCS) and a non-related peptide were synthesized on cellulose membrane by spot-synthesis. Figure 2 shows the specific reactivity of serum, demonstrating the recognition when samples from *L. infantum* infected animals were used.

### ELISA after cycles of immunization

**Figure 3:**
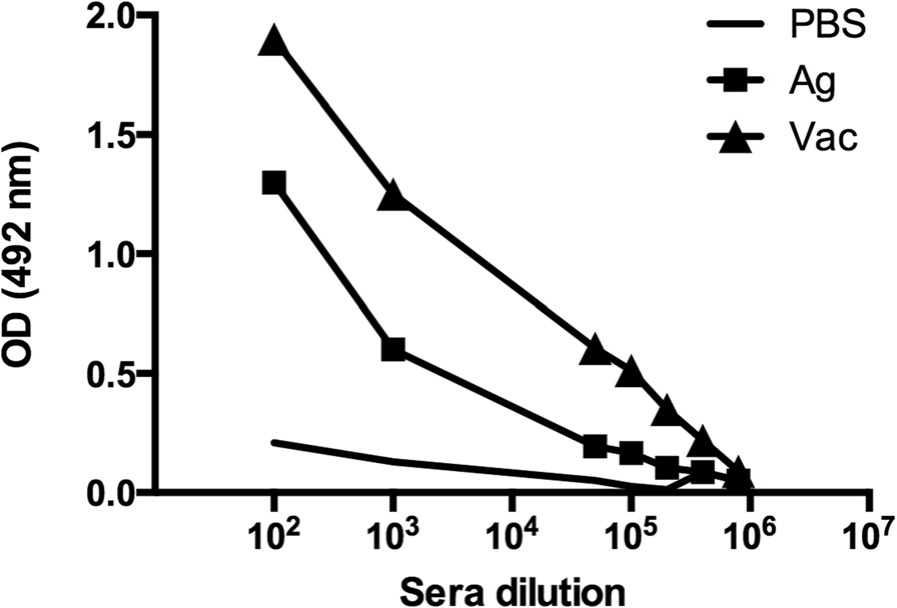
**Immune response in peptide-immunized mice evaluated by ELISA.** Sera were serially diluted from 1 to 100 and up to 10^7^. Pre-immune sera were used as negative control. The data are expressed as line graphs (Phosphate Buffered Saline, PBS = line; *Leishmania infantum* antigen, Ag = black squares; Peptide 5, Vac = black triangles).

One week after last immunization, antibody levels in mice serum were analyzed by Indirect ELISA. Figure 3 shows absorbance values in animals immunized with Peptide 5, LiAg and PBS. Vaccination with Peptide 5 and LiAg elicited significant antigen-specific IgG responses compared to PBS. Antibodies titers were 1:400,000 (Peptide 5 group/Vac) and 1:100,000 (LiAg group/Ag).

### Cytokines production assay

**Figure 4:**
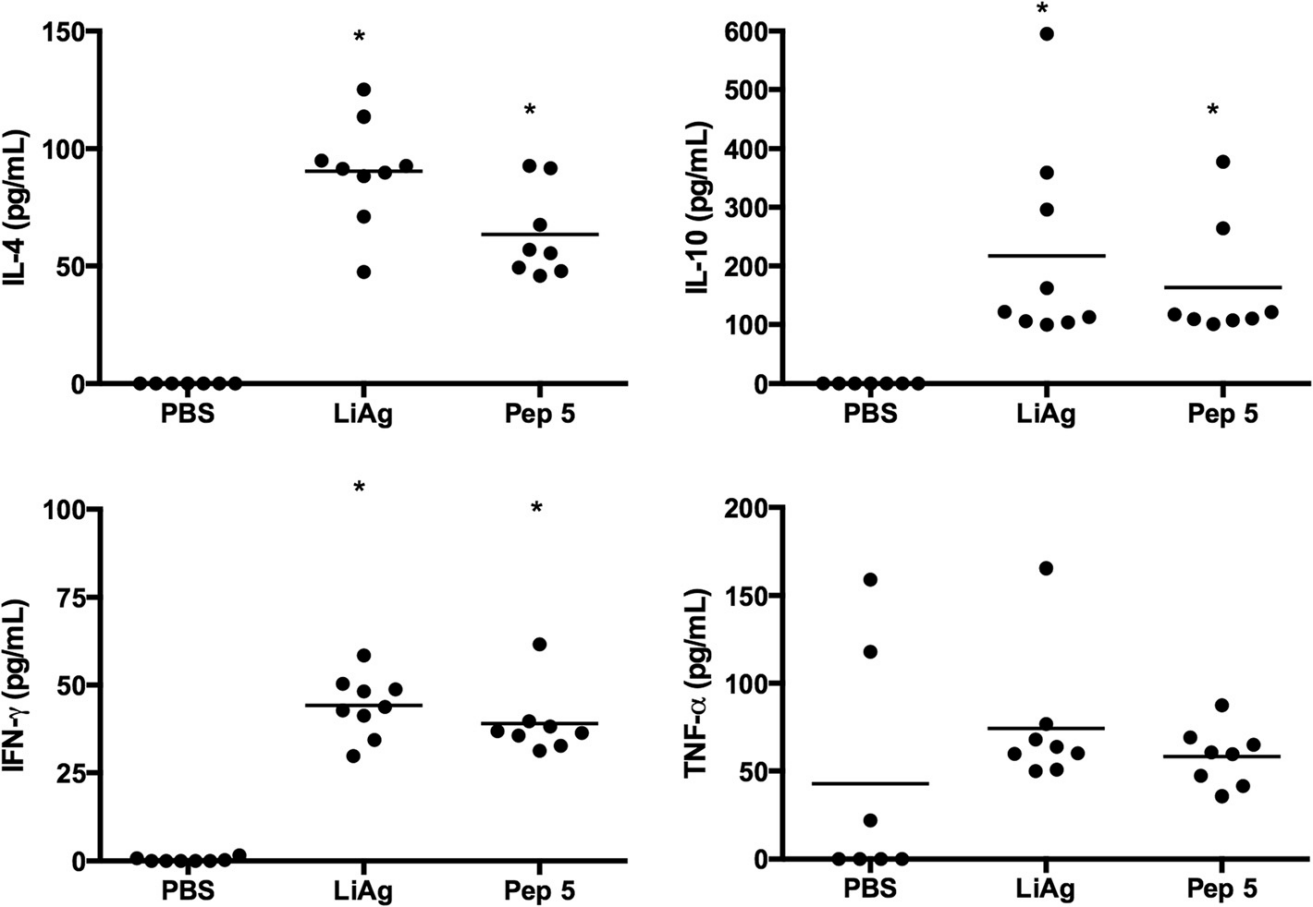
**Cytokine levels detected in culture supernatants of murine PBMC.** Levels of IFN-γ, TNF-α, IL-10 and IL-4 detected in PBMC culture supernatants of three groups (PBS, LiAg and Pep 5) produced in response to LiAg stimulation. The results are expressed as scattering of individual values (black circles) and mean of that group (line). Statistically significant differences in the parasite load between experimental groups are showed in the graph by “*” symbol.

We determined whether immunization with Peptide 5 resulted in increased IFN-γ or IL-10 production in response to LiAg stimulation. As shown in Figure 4, PBMC from mice vaccinated with peptide 5, before infection, secreted significantly higher levels of IFN-γ when stimulated with LiAg than PBMC collected from control infected mice. Additionally, the production of IL-10 was not significantly higher in the vaccinated mice as compared to control animals. No cytokine production was observed when cells were stimulated with Peptide 5 alone.

### Parasite load by quantitative PCR

**Figure 5:**
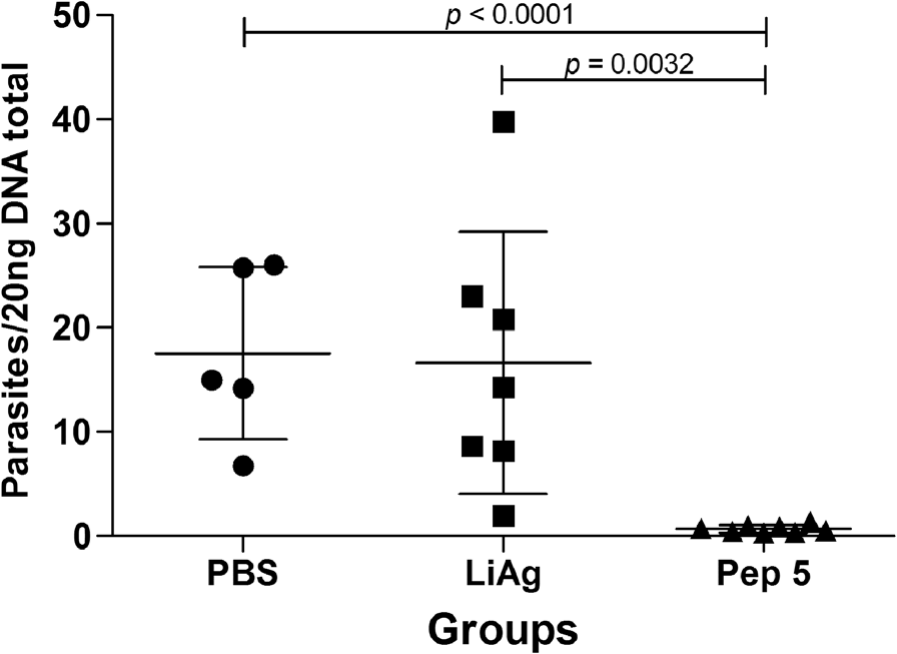
**Parasite load after challenge infection.** The number of parasites in the spleen was measured by Real-Time PCR technique and showed by number of parasites *per* 20 ng of DNA total. DNA was extracted from the spleen samples of mice immunized with vaccines containing Phosphate Buffered Saline (PBS = black circles - control group), *Leishmania infantum* antigen (LiAg = black squares) or Peptide 5 (Pep 5 = black triangles). Mean ± standard deviation (SD) in each group is shown. Statistically significant differences in the parasite load between experimental groups are showed in the graph.

Parasite quantification was performed using DNA extracted from the spleen samples of mice from Peptide 5, LiAg and control groups. Animals immunized with Peptide 5 presented significant reduction of parasite burden (up to 98%), when compared to control and LiAg groups (Figure 5). Peptide 5 demonstrated an excellent effectiveness as vaccine antigen.

## Discussion

Development of a protective vaccine in dogs is the best strategy to efficiently control CVL, promoting a possible reduction of infectivity to sand fly vectors and, consequently, the transmission to humans [27]. A practical vaccine for use should be safe, inexpensive and elicit long-lasting immunity [28]. The use of synthetic peptides in vaccine field is promising [29]; when compared to the production of whole proteins, peptides are simpler to synthesize and are cheaper to produce [30]. The success of a vaccine depends on our current understanding of the characteristics of an effective anti-*Leishmania* immune response, as they have been already determined from human and murine studies [31,32].

In the present work, the selection of a leishmanial immunodominant epitope was performed by Phage Display technique. Sequence analysis in the GenBank databases did not reveal any significant similarity to the amino acid sequence of antigens previously characterized from *Leishmania*, suggesting this sequence corresponds to either unknown proteins or to mimotopes (conformational epitopes of *Leishmania* proteins). Furthermore, we demonstrated that immunization of mice with peptide 5 formulated with aluminum hydroxide and liposomes rendered high levels of antibodies, suggesting the immunogenicity was conferred by combined delivery technique and formulation with adjuvants, as immunogenicity might be improved after appropriate use of adjuvants [33]. The use of a proper adjuvant constitutes an important aspect in obtaining an efficient protocol of immunization [27]. Since the 1970s, it has been known that liposomal presentation of antigens can confer greater immunogenicity when compared to antigen alone [34,35]. Some advantages of liposomal vaccines are an opportunity for dose-sparing of antigen, plasticity with regard to lipid composition and recruitment of various components of the immune system [36,37]. In addition, liposomes also provide a physical means for either delivering encapsulated antigens or presenting surface-associated antigens with the ability to modulate epitope density and homogeneity.

Our findings demonstrated that experimental vaccine formulation (Peptide 5, liposome and Aluminum Hydroxide) achieved high levels of protection and produced a mixed Th1/Th2 response in mice. Briefly, resistance to leishmaniasis has been associated with a predominant IFN-γ production from the antigen-specific CD4+ T lymphocyte population-termed T helper 1 (Th1) immune response [38]. These cells might be then effective in promoting macrophage activation at the site of the lesion, and the intracellular *Leishmania* are killed in a nitric oxide–dependent manner. Interestingly, while the protection conferred by Peptide 5 was significantly higher than observed in animals immunized with crude leishmanial antigen, the cytokine profile induced by immunization was similar in both groups. These data suggest that achievement of protection against *L. infantum* might also depend on other factors rather than solely in the cytokine production.

Concerning the vaccine formulation, Aluminum-containing adjuvants are often used in vaccines against infectious diseases and in preparations for allergy immunotherapy [39,40]. However, they selectively stimulate a Th2 immune response in mice and a mixed response in human beings [41]. On the other hand, adsorption of antigens to aluminum adjuvants enhances the immune response by increasing phagocytosis and slowing the diffusion of antigens from the injection site, which allows larger time for inflammatory cells. The adsorptive strength is important as high affinity interactions interfere with the immune response. Adsorption is also associated to the physical and chemical stability of antigens [42-44].

While the use of vaccine using Peptide 5 trapped in sphingomyelin/cholesterol liposomes conferred a considerable degree of protection in the employed experimental model it is not clear whether protection is associated with long-term immunity considering the time between immunizations and challenge infection. Further studies are required to determine the profile of memory responses and extent of immunogenicity after immunization process. However, despite the possibility that vaccination with Peptide 5 might induce extrafollicular reaction (thus generating short-lived B cells) [45] or production/expansion of effector T cells [46] – highly effective against the pathogen but with limit duration of immunity, the use of such formulation may be considered as a component of future multivalent vaccines due to a high degree of protection even a few days after immunization. Moreover, further studies are also required to describe the impact of different adjuvants regardless of the evidence that liposomes represent an improvement of vaccine formulation [47]. Promising findings obtained in the present study encourage canine trials to confirm the effectiveness of a vaccine against CVL, which will control and reduce the chances of infectivity to sand fly vectors and consequently the transmission to dogs and humans.

## Conclusions

Taken together our findings demonstrated that immunization with vaccine using the synthetic peptide 5 trapped in sphingomyelin/cholesterol liposomes achieved high levels of protection against visceral leishmaniasis and elicited a mixed Th1/Th2 response in mice. These results encourage its further use in canine trials to confirm the effectiveness of a vaccine against CVL.
